# Downregulation of Low-density lipoprotein receptor-related protein 1B (LRP1B) inhibits the progression of hepatocellular carcinoma cells by activating the endoplasmic reticulum stress signaling pathway

**DOI:** 10.1080/21655979.2022.2060778

**Published:** 2022-04-07

**Authors:** Zili Zhen, Zhemin Shen, Peilong Sun

**Affiliations:** a Department of General Surgery, Jinshan Hospital, Fudan University, Shanghai, P. R. China; b Department of Surgery, Shanghai Medical College, Fudan University, Shanghai, P. R. China; c Center for Tumor Diagnosis and Therapy, Jinshan Hospital, Fudan University, Shanghai, P. R. China

**Keywords:** *LRP1B*, hepatocellular carcinoma, progression, doxorubicin, PERK-ATF4-CHOP pathway, prognosis

## Abstract

Hepatocellular carcinoma (HCC) has a high recurrence rate and mortality rate even after surgery. Low-density lipoprotein receptor-related protein 1B (LRP1B) has proven to be involved in tumor development and progression of multiple malignancies. However, the function of *LRP1B* in HCC progression has not been fully elucidated. Thus, we conducted this study to explore the relationship between *LRP1B* and HCC. Bioinformatic analyses implied that *LRP1B* was highly expressed in HCC tissues. High *LRP1B* expression was shown to be related to poor outcomes and the determination of HCC patients’ tumor stage. *LRP1B* deletion impeded the proliferation, migration, and invasion of HCC cells. Further investigation demonstrated that silencing *LRP1B* expression enhanced the sensitivity of HCC cells to doxorubicin. *LRP1B* deletion inhibited HCC progression by regulating the PERK-ATF4-CHOP signaling pathway. Additionally, we probed the genomic alterations of *LRP1B* in HCC and the impact on the prognosis of patients. Collectively, our results suggest that *LRP1B* plays an essential role in the promotion of HCC progression by regulating the PERK-ATF4-CHOP signaling pathway, which is a potential prognostic biomarker and a promising therapeutic target of HCC.

## Graphical abstract

**Figure:**
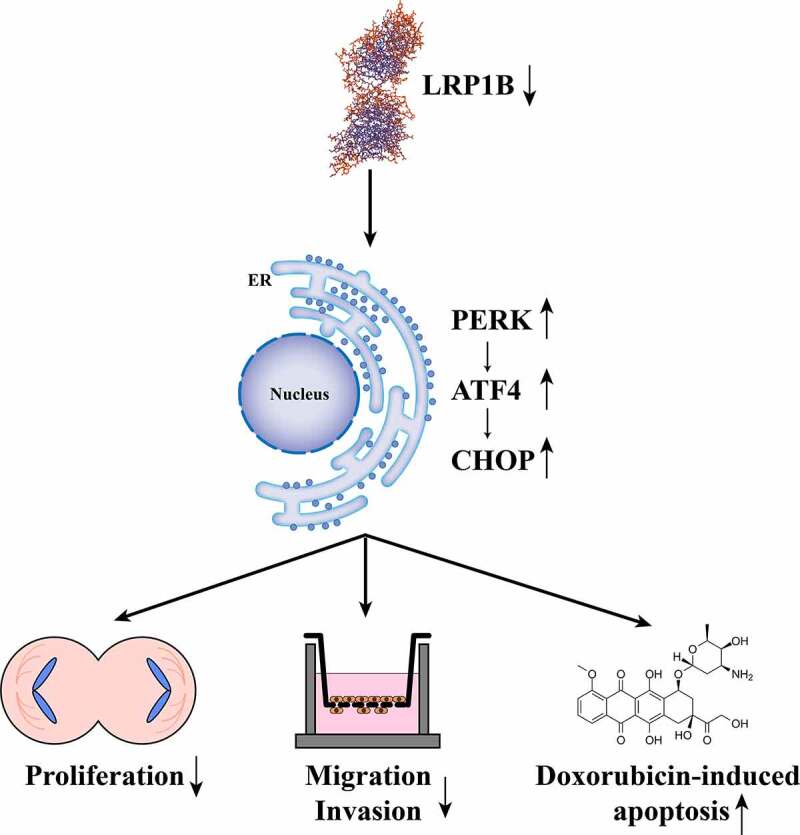

## Introduction

Primary liver cancer ranks sixth for common malignant tumors incidence globally, with its mortality rate ranking third in cancer death [1]. Hepatocellular carcinoma (HCC) is the most common type of liver cancer, accounting for approximately 80% of cases [2]. The reduction in the incidence of HCC is due to the popularization of the hepatitis B vaccine and the development in terms of diagnosis and treatment. For patients with advanced HCC or poor liver function, however, the existing treatment modality is still unable to reduce the mortality rate effectively [3]. Meanwhile, some first-line targeted therapy drugs used for systemic treatment of patients with HCC, such as doxorubicin, are encountering chemotherapy resistance problems that lead to poor prognosis [4,5]. Therefore, there is a tremendous and urgent need to identify novel biomarkers for HCC.

Low-density lipoprotein receptor-related protein 1B (LRP1B) is a member of the LDLR protein family, with various biological functions, including cell signaling and cargo transport [6,7]. Increasing attention has been directed toward the role of *LRP1B* in the field of cancer research, that being, its involvement in proliferation, metastasis, angiogenesis, and differentiation in multiple cancers [8–11]. Moreover, previous studies have suggested a close relationship between *LRP1B* mutations and tumor mutational burden (TMB), which can predict prognosis and therapy targets in cancer patients [12]. Notably, the *LRP1B* mutation is associated with a worse prognosis in HCC patients, displaying relation to TMB and immune infiltration [13,14]. Nevertheless, the expression status and potential functions of *LRP1B* in cancer are yet to be unveiled, especially in HCC.

In this study, we evaluated the expression level of *LRP1B* in HCC and the correlations between *LRP1B* mRNA expression and other clinical features. *LRP1B* was highly expressed in HCC tissue and was not conducive to prognosis. We assumed that *LRP1B* might play a tumor-promoting role in HCC progression. We aimed to explore the function and mechanism of *LRP1B* in HCC cells proliferation, migration, invasion, and drug sensitivity by *LRP1B* knockdown. Another goal was to investigate the genomic mutation of *LRP1B* and its relationship with prognosis.

## Materials and methods

### Cell culture

The human normal liver cell line LX-2 was purchased from Shanghai Zhong Qiao Xin Zhou Biotechnology Co.,Ltd (Shanghai, China) whereas the human HCC cell lines HepG2 and HuH-7 were bought from the Cell Bank of the Institute of Biochemistry and Cell Biology of the Chinese Academy of Sciences (Shanghai, China). LX-2, HepG2, and HuH-7 were cultured in Dulbecco’s modified Eagle’s medium (KeyGEN, China), supplemented with 10% fetal bovine serum (FBS; Gibco, United States) in a humidified incubator containing 5% CO2 at 37°C.

### Cellular transfection

The specific *LRP1B*-small interfering RNA (siRNA) and negative control (NC)-siRNA were synthesized by Genepharma (Shanghai, China). The sequences of siRNA are listed in Table S1. According to the manufacturer’s instructions, Lipofectamine 3000 (Invitrogen, United States) was used for transfection. Upon the cells’ attainment of a 60% confluency, 4 μL siRNA and 4 μL Lipofectamine 3000 Reagent were added to100μL Opti-MEM Reduced Serum Medium (Gibco, United States), respectively. After 10 min of preparation, the two reagents were mixed and incubated at room temperature for 10 min. The cells were cultured in a serum-free medium that was supplemented with the mixture. HepG2 and HuH-7 cells were cultivated in a serum-free medium with *LRP1B*-siRNA or NC-siRNA for 12 hours. Thereafter, the medium was substituted with a complete medium. Real‐time polymerase chain reaction (RT-PCR) was conducted to test the efficiency of *LRP1B* knockdown.

### RNA extraction and RT-PCR

Total RNA in the cells was extracted by using an RNA-Quick Purification Kit (Yishan Biotechnology Co., Ltd, China). We evaluated the quality of the extracted total RNA by A260/A280 absorption (1.9 ~ 2.2). RT-PCR assays were performed as previously described [15]. We calculated the relative expression of target genes by utilizing the 2^−ΔΔCt^ method. The specific primer sequences of GAPDH and LRP1B are displayed in Table 1.

**Table 1.** The specific primer sequences for RT-PCR

Gene symbol	Sequence (5’ → 3’)
**LRP1B**	
Forward primer	CCGGAATACACCGGAGACAG
Reverse primer	CCCATGGCACCTTACACACT
**GAPDH**	
Forward primer	GGAGCGAGATCCCTCCAAAAT
Reverse primer	GGCTGTTGTCATACTTCTCATGG

### Protein extraction and Western blot analyses

We lysed cells with SDS Lysis Buffer (Beyotime, China), containing 1% protease inhibitor (Beyotime, China) and 1% phosphatase inhibitor (KeyGEN, China). The lysates were then sonicated and pelleted by centrifugation. We measured protein concentrations using the BCA protein assay kit (KeyGEN, China). According to previous reports, Western blot analysis was performed on lysates of equal mass [16]. The following antibodies were utilized for immunoblotting: N-cadherin (13116S, 1:2000), E-cadherin (14472S, 1:1000), vimentin (5741S, 1:2000), Bcl-2 (4223S, 1:1000), Bax (14796S, 1:2000), p53 (2527S, 1:2000), PERK (5683S, 1:2000), ATF4 (11815S, 1:1000), CHOP (2895S, 1:1000), BiP (3177S, 1:2000), β-actin (4970S, 1:5000) from Cell Signaling Technology (United States), and LRP1B (46,033, 1:1000) from Signalway Antibody (United States).

### Cell counting kit-8 (CCK-8) assays

HepG2 and HuH-7 cells were grown on 96-well plates at a density of 5000 cells per well and transfected with *LRP1B*-siRNA or NC-siRNA. After culturing for 12, 24, 36, 48, 72 hours in a 37°C incubator, CCK-8 (KeyGEN, China) was added to the cells to facilitate detection of the optical density at 450 nm with a microplate reader (BioTek, United States).

### EdU assays

HepG2 and HuH-7 cells were plated in 24-well plates with a density of 20,000 cells per well. After transfection of *LRP1B*-siRNA or NC-siRNA, they were cultured at 37°C for 48 hours and then processed with an EdU Cell Proliferation Kit with Alexa Fluor 488 (Epizyme, China) [17]. Concisely, the cells were incubated with EdU for 4 hours, fixed with 4% paraformaldehyde for 15 min, and penetrated with Triton X-100 for 10 min. Immediately afterward, the cells were incubated at room temperature in a dark place with the Click Reaction Mixture for 30 min and then with Hoechst 33,342 for 10 min.

### Colony formation assays

HepG2 and HuH-7 cells transfected with *LRP1B*-siRNA or NC-siRNA were seeded in six-well plates with a density of 1000 cells/well. After culturing for one week, the cells were fixed with 4% paraformaldehyde and stained with crystal violet staining solution. Image J was utilized for the count of clone formation.

### Wounding healing assays

With a density of 200,000 cells/well, HepG2 and HuH-7 cells were seeded in a six-well plate. After completion of the transfection and the cell confluence’s attainment of 90%, the cells were scratched with a 200 μL pipette tip. After the separated cells were washed and removed with PBS, they were cultured in a 37°C incubator with a serum-free medium. The wound closure process was observed under a microscope at 12, 24, 36, 48, and 72 hours. The wound area was calculated with Image J.

### Transwell assays

To test the invasive ability of HCC cells, Matrigel matrix (final concentration 250 µg/ml; Corning, United States) was first added to cover the upper chamber of the Transwell before the cells were incubated [18]. In contrast, the cells were directly added to the upper chamber in the migration experiment. The HepG2 and HuH-7 cells transfected with *LRP1B*-siRNA or NC-siRNA were added to the upper chamber at a density of 50,000 cells per well, cultured with serum-free medium, and 600 μL medium containing 20% FBS was added to the lower chamber. The cells that migrated and invaded through the membrane were stained with 4% paraformaldehyde and crystal violet staining solution 48 hours later. An optimal microscope was utilized to photograph the cells whereas Image J was used to count the number of migrated and invaded cells.

### Apoptosis detection

After transfection of *LRP1B*‐siRNA or NC-siRNA, the cells were cultured in a six-well plate for 48 hours. The cells were washed with PBS, harvested with EDTA-free trypsin, and stained in accordance with Annexin V Apoptosis Detection Kit’s instructions (Dojindo, Japan). Incubation for 15 min in the dark at room temperature was followed by measuring of the apoptotic cells by flow cytometry (Beckman Coulter, Inc, United States) [19].

### Detection of sensitivity of HCC cells to doxorubicin

The toxicity of doxorubicin (HY-15142; MedChemExpress, United States) was detected by CCK-8, and the sensitivity of cells to doxorubicin was assessed using the half-maximum inhibitory concentration (IC50) [20]. HepG2 and HuH-7 cells were seeded in a 96-well plate at a density of 5000 cells/well and transfected. The cells were treated with 0, 0.0625, 0.125, 0.25, 0.5, 1 μM doxorubicin for 48 hours, after which the absorbance confirmed the cell viability at 450 nm.

### Transcriptome and somatic mutation data collection

HCC transcriptomic data were acquired from The Cancer Genome Atlas (TCGA) and the Gene Expression Omnibus (GEO) dataset (GSE45114 and GSE164760) [21,22]. Somatic gene mutation data of HCC samples from the United States and China were collected from TCGA and the International Cancer Genome Consortium (ICGC), respectively. Besides, the TCGA portal also acted as a source for the obtainment of corresponding clinical data. The specific information of the datasets is summarized in Table S2.

### Prognostic value analysis

Based on the median value of *LRP1B* as a cutoff, the HCC patients in the TCGA dataset were subdivided into two groups (high vs. low expression). To assess the prognostic value of *LRP1B*, we applied the R ‘survival’ package for the Kaplan-Meier (K-M) survival analysis to compare the differences in overall survival (OS), progression-free interval (PFI), disease-free interval (DFI), and disease-specific survival (DSS) between the two groups. Similarly, we grouped HCC patients based on the presence and absence of *LRP1B* mutation (wild-type vs. mutation) and performed the survival analysis.

### Gene set enrichment analysis (GSEA)

The gene with a different prognosis either caused by mutation or not was utilized for the enrichment analysis by gene set ‘c2.cp.v7.2.symbols.gmt’ [23]. Permutations were set to 1000 to obtain a normalized enrichment score. The normalized p-value and false discovery rate (FDR) were acquired for each gene set enrichment. The criterion for significant enrichment is a normalized p-value < 0.05.

### Statistical analysis

R v 4.0.1 or GraphPad Prism v 8.0 (GraphPad Software, Inc, United States) was used to analyze all data. Statistical significance was achieved with a p-value < 0.05. Comparisons between two groups were carried out by either the Student’s t-test or the Wilcox test where appropriate. We constructed survival curves using K-M analysis and then compared them with the two-tailed log-rank test.

## Results

A high expression of *LRP1B* was found in HCC tissues and cells by bioinformatics, RT-PCR, and Western blot analyses. *LRP1B*’s high expression was not conducive to the prognosis of HCC patients. The effect of *LRP1B* on the proliferation, migration, invasion, and doxorubicin sensitivity of HCC cells by *LRP1B* knockdown was then investigated. The downregulation of *LPR1B* suppressed HCC progression by activating the endoplasmic reticulum stress signaling pathway. In addition, we also clarified the effect of *LRP1B* mutation on the prognosis of HCC patients.

### LRP1B *is highly expressed in HCC*

**Figure 1.**
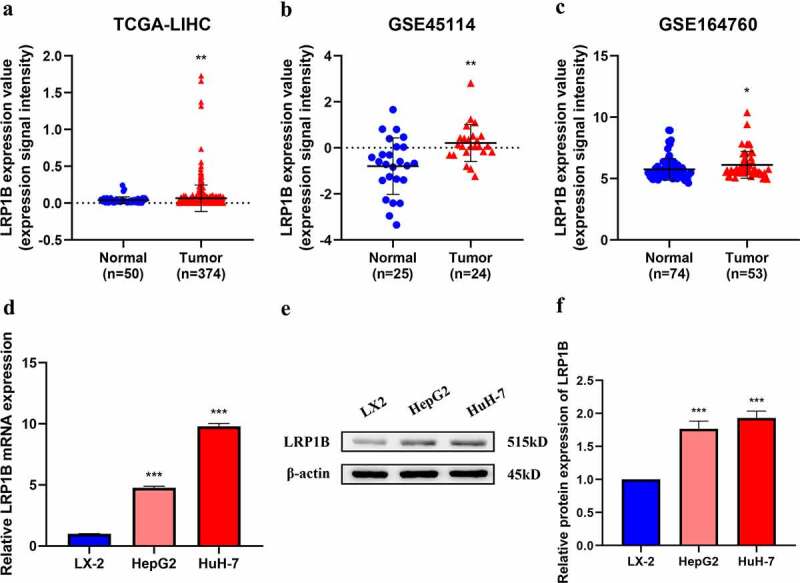
**LRP1B expression profile in HCC**. (a-c) Comparison of LRP1B mRNA expression between normal liver tissues (Normal) and HCC tissues (Tumor). Data were obtained from three gene expression datasets (TCGA-LIHC, GSE45114, and GSE164760). (d) Relative LRP1B mRNA expression of hepatoma cells and HCC cell lines by RT‐PCR. (e-f) Relative LRP1B protein expression of hepatoma cells and HCC cell lines by Western blot. *p < 0.05, **p < 0.01, ***p < 0.001.

HCC transcriptome datasets from TCGA and GEO were utilized in the examination of the landscape of *LRP1B* expression in HCC tissues to perform a differential expression analysis of the gene. HCC tissues expressed *LRP1B* at significantly higher levels than the normal adjacent tissues in TCGA-LIHC, GSE45114, and GSE164760 (Figure 1a-c). A detection of *LRP1B* expression in three different cell lines demonstrated it to be upregulated in HCC cell lines (HepG2 and HuH-7) compared with that of normal liver cell lines (LX-2) by RT-PCR analysis (Figure 1d). Western blot showed that the protein level of LRP1B was higher in HepG2 and HuH-7 cells than in LX-2 cells (Figures 1e, f).

### *Correlation between* LRP1B *expression level and the clinical characteristics of HCC patients*

**Figure 2.**
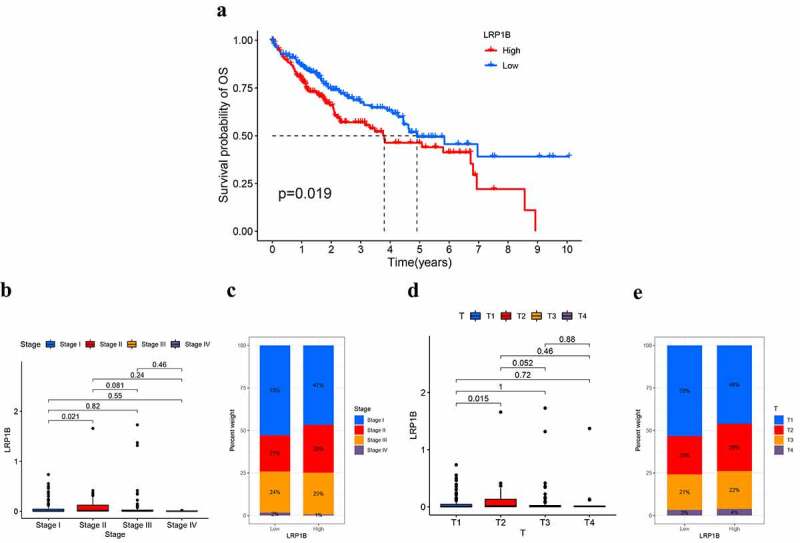
**The correlation between the expression of LRP1B and clinical characteristics**. The K-M survival curves for OS (a) of HCC with low (below median) or high LRP1B (above median). The association of LRP1B mRNA expression with the tumor stage (b) and T classification (c) in the TCGA cohort. The proportion of tumor stages (d) and T classification (e) in different expressions of LRP1B.

A total of 371 HCC patients from the TCGA dataset were involved in the study to explore the correlation between clinical characteristics, such as age, gender, prognosis, histological grade, TNM stage, cancer status, and *LRP1B* expression. Patients were divided into two groups according to the median expression levels of *LRP1B*. K-M analysis was used for the examination of the association between the prognosis and the *LRP1B* expression, revealing a significant discrepancy in OS between the two groups, but not in PFI, DFI, or DSS (Figure 2a, Figure S1). The 1-, 3-,5-year survival rates in the high expression group were 79.2%, 55.5%, 44.0%, respectively, while the corresponding values were 86.0%, 67.4%, 49.4% in the low expression group. Compared to patients in stage I, the expression level of *LRP1B* was significantly higher in stage II patients (Figure 2b, c). Similarly, a similar trend was displayed by the difference in the expression of *LRP1B* in HCC tissues between T2 and T1 (Figure 2d, e). These findings suggested that *LRP1B* might contribute to HCC development. There were no other differences in *LRP1B* expression between the groups in the listed characteristics (Figure S2).

### LRP1B *depletion suppresses HCC cells proliferation*

**Figure 3.**
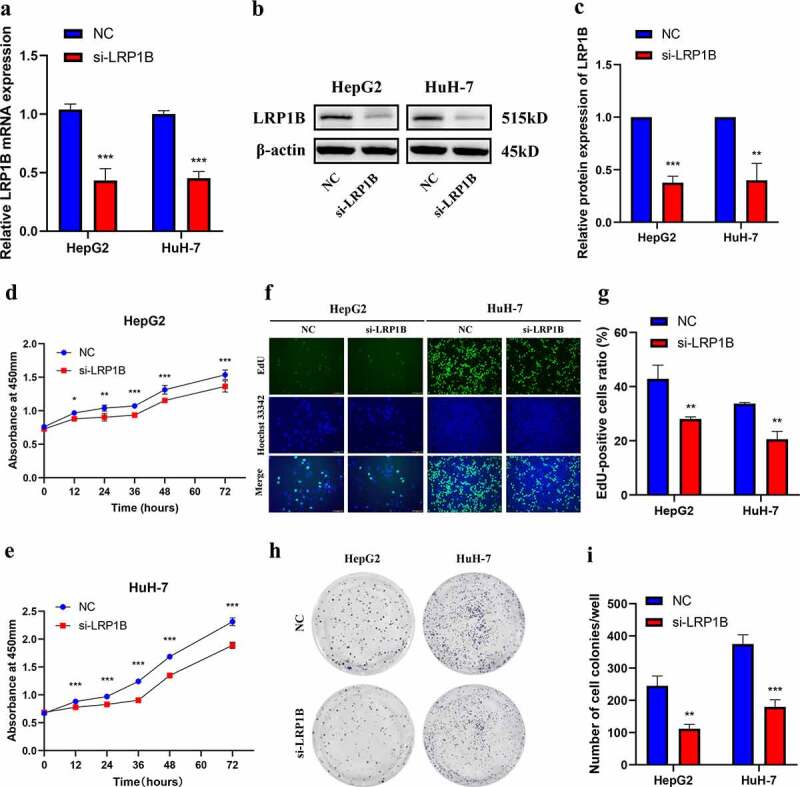
**Effect of LRP1B regulates HCC cell proliferation**. (a) Knockdown of LRP1B efficiency determined by RT‐PCR in HepG2 and HuH-7 cells. (b-c) Knockdown of LRP1B efficiency determined by Western blot analysis in HepG2 and HuH-7 cells. (d-e) Detection of HepG2 and HuH-7 cell proliferation after LRP1B knockdown using the CCK‐8 assay. (f-g) Detection and quantification of EdU-positive HepG2 and HuH-7 cells after LRP1B knockdown (h-i) Colony formation capacity of LRP1B knockdown in HepG2 and HuH-7 cells. *p < 0.05, **p < 0.01, ***p < 0.001.

To explore the function of *LRP1B*, we designed the specific siRNA (si-*LRP1B*) inhibiting *LRP1B* expression. *LRP1B* knockdown led to a 40–60% reduction in the *LRP1B* expression based on Western blot and RT-PCR in HepG2 and HuH-7 cells (Figure 3a-c). The CCK-8 assays showed a decreased proliferation rate of HepG2 and HuH-7 cells due to *LRP1B* knockdown (Figure 3d, e). The EdU staining demonstrated similar results, wherein the knockdown of *LRP1B* resulted in fewer EdU‐positive HCC cells. This suggested that *LRP1B* knockdown decreased HCC cells proliferation compared with NC cells (Figure 3f, g). Moreover, the colony formation assays were conducted to assess the effects of *LRP1B* on the biological behaviors of these HCC cells. The clonogenic capacity of *LRP1B* knockdown cells was significantly impaired compared to NC cells (Figure 3h, i). In short, these results demonstrated that *LRP1B* was crucial for HepG2 and HuH-7 cells proliferation and colony formation.

### *Knockdown of* LRP1B *reduces migration and invasion in HCC cell lines*

**Figure 4.**
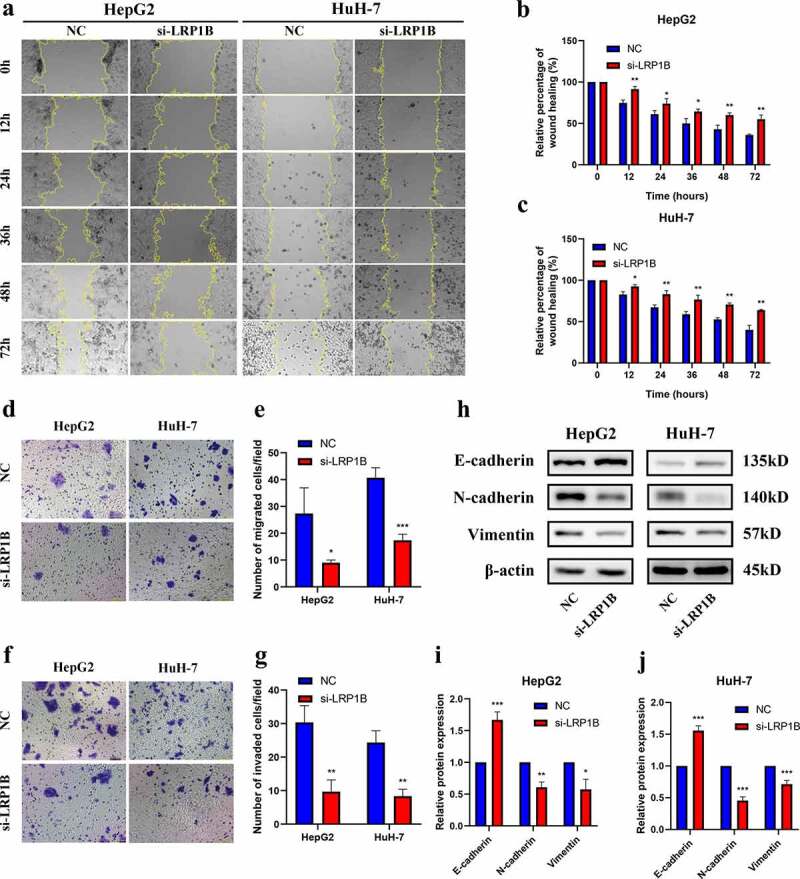
**Effect of LRP1B regulates HCC cell migration and invasion**. (a-c) Detection of cell migration ability after LRP1B knockdown using the wound healing assay. (d-e) Transwell migration assay in HepG2 and HuH-7 cells with LRP1B knockdown. (f-g) Transwell invasion assay in HepG2 and HuH-7 cells with LRP1B knockdown. (h-j) Western blot analyses of proteins related to EMT in HepG2 and HuH-7 cells with LRP1B knockdown. *p < 0.05, **p < 0.01, ***p < 0.001.

The wound-healing assays were employed to test the migration ability in HCC cells after transfected with si-*LRP1B*. Both HepG2 and HuH-7 cells with si-*LRP1B* transfection showed weaker cellular migration than NC transfection after 12 hours (Figure 4a-c). Similarly, the migration ability of si-*LRP1B* transfected cells was reduced as observed by the Transwell migration assay (Figure 4d, e). The Transwell Matrigel invasion assay indicated that the invasive capability of HCC cells after si-*LRP1B* transfection was also markedly weakened (Figure 4f, g). Additionally, the level of hub protein related to epithelial-mesenchymal transition (EMT) was determined by Western blot analysis, displaying an increase in the E-cadherin level but a decrease in the N-cadherin and vimentin levels in *LRP1B* knockdown cells (Figure 4h-j). The results mentioned above demonstrated that the cell migratory and invasive capabilities of HCC cells were significantly reduced in *LRP1B* knockdown cells.

### *Silencing* LRP1B *increases the sensitivity to doxorubicin in HCC cells*

**Figure 5.**
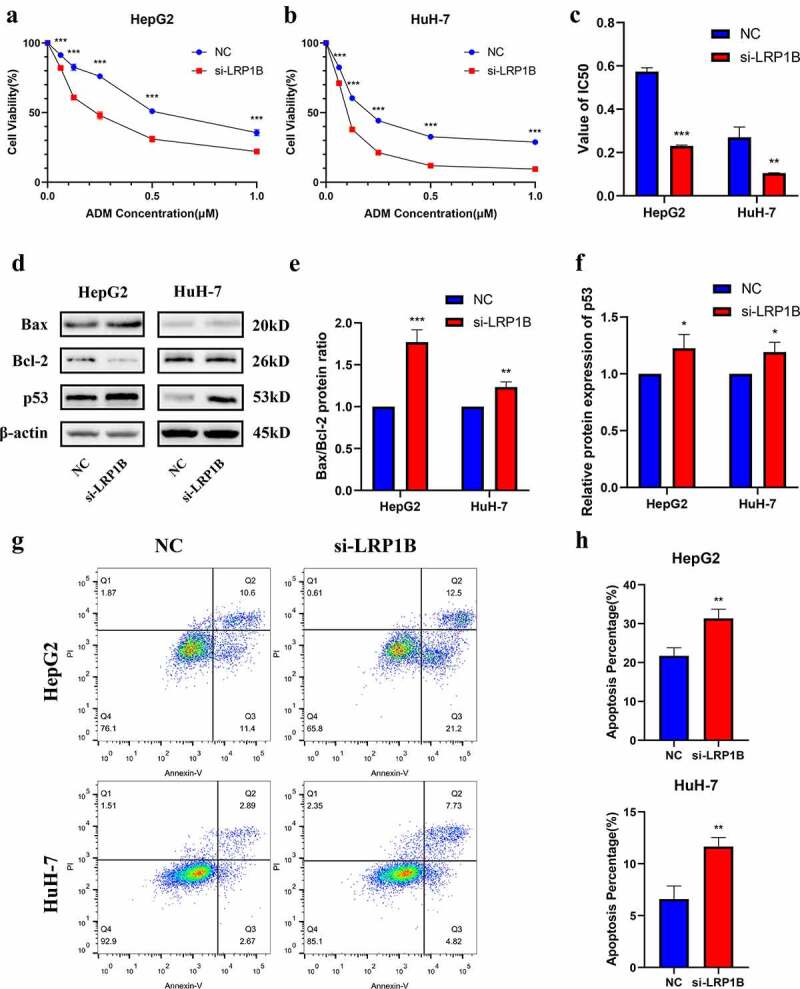
**Effect of LRP1B knockdown on HCC cell sensitivity to doxorubicin**. (a-b) CCK‐8 assay was used to test the effects of doxorubicin on the viability of HepG2 and HuH-7 cells with LRP1B knockdown. Cells were treated with increasing doxorubicin concentrations for 48 hours. (c) Statistic analyses of IC50 values of LRP1B knockdown HepG2 and HuH-7 cells treated with doxorubicin. (d-f) Western blot results of proteins related to apoptosis in LRP1B knockdown HepG2 and HuH-7 cells treated with doxorubicin. (g-h) Flow cytometry results of apoptosis in LRP1B knockdown HepG2 and HuH-7 cells with doxorubicin. ADM: doxorubicin, *p < 0.05, **p < 0.01, ***p < 0.001.

There was no apparent change in apoptosis after *LRP1B* deletion in HepG2 and HuH-7 cells (Figure S3). CCK-8 assays were utilized to detect whether *LRP1B* affected the drug sensitivity of HCC cells to doxorubicin. With the increase of drug concentration, the viability of HepG2 with *LRP1B* knockdown was significantly lower than that of NC cells while analogous results were observed in HuH-7 cells (Figure 5a, b). Indeed, the IC50 value of HepG2 and HuH-7 cells following doxorubicin treatment also declined with *LRP1B* deletion (Figure 5c, Figure S4). Moreover, the Western blot was used to detect the checkpoint proteins of apoptosis induced by doxorubicin. The Bax/Bcl-2 ratios and the protein expression of p53 were elevated in both HepG2 and HuH-7 cells with *LRP1B* knockdown (Figure 5d-f). The results of flow cytometry also showed that *LRP1B* knockdown led to a significant increase in apoptosis of HepG2 and HuH-7 cells with doxorubicin (Figure 5g, h). These results suggested that *LRP1B* silencing effectively increased the sensitivity of HepG2 and HuH-7 cells toward doxorubicin through the main mechanism of enhancing doxorubicin-induced apoptosis.

### LRP1B *depression regulates the PERK-ATF4-CHOP pathways*

**Figure 6.**
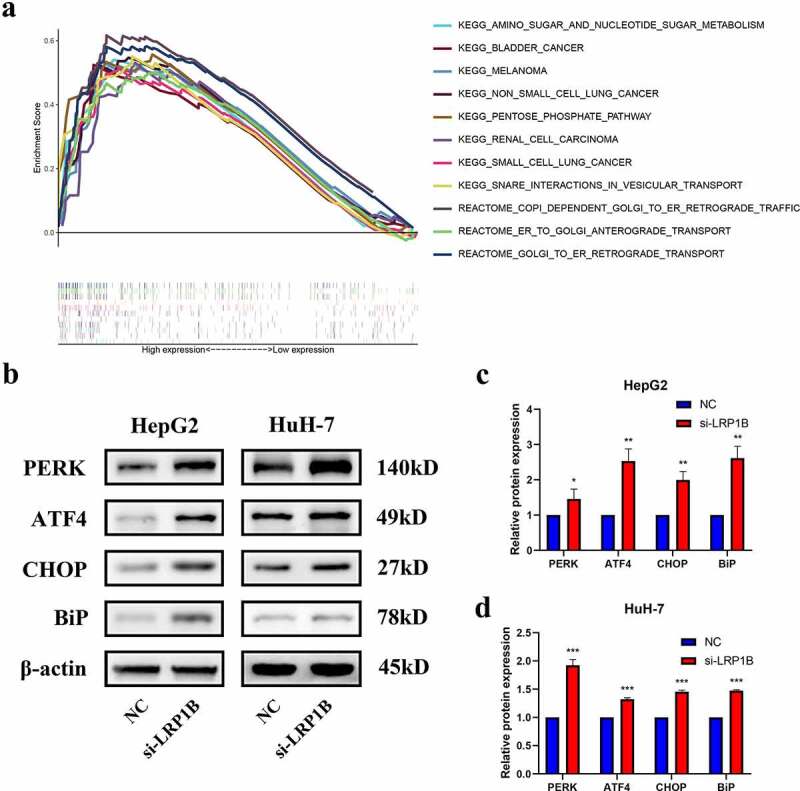
**The protein expression level of PERK-ATF4-CHOP signaling pathway after LRP1B knockdown**. (a) GSEA analysis of HCC samples with different LRP1B expression from the TCGA cohort. Analyses of PERK, ATF4, CHOP, and BiP protein expression as shown in the western blots (b) and quantified levels (c-d). *p < 0.05, **p < 0.01, ***p < 0.001.

Growing evidence has indicated that endoplasmic reticulum stress plays an essential role in tumor progression. GSEA enrichment analysis showed that HCC samples with high *LRP1B* expression were significantly enriched in endoplasmic reticulum-related pathways, such as COPI Dependent Golgi to ER Retrograde Traffic, ER to Golgi Anterograde Transport and Golgi to ER Retrograde Transport (all normalized p-value < 0.05) (Figure 6a). Moreover, some other malignant tumor pathways and tumor metabolic pathways were also enriched (all normalized p-value < 0.05). The interplay between *LRP1B* and the endoplasmic reticulum stress pathway in HCC was studied through examination of the expression of molecular markers of endoplasmic reticulum stress by Western blot analysis, such as PERK, ATF4, CHOP, and Bip. Here, PERK-ATF4-CHOP represents a classical endoplasmic reticulum stress pathway while Bip describes an endoplasmic reticulum-resident chaperone. In HepG2 cells, *LRP1B* knockdown caused a dramatic increase in PERK, ATF4, CHOP and Bip protein expression in comparison to that of the NC group (Figure 6b, c). Consistently, the Western blot analysis confirmed similar results in *LRP1B* knockdown HuH-7 cells (Figure 6b, d). In sum, our data strongly suggested that downregulation of *LRP1B* retard HCC tumor progression through the PERK-ATF4-CHOP signaling.

### *Genomic alterations of* LRP1B *are associated with HCC*

**Figure 7.**
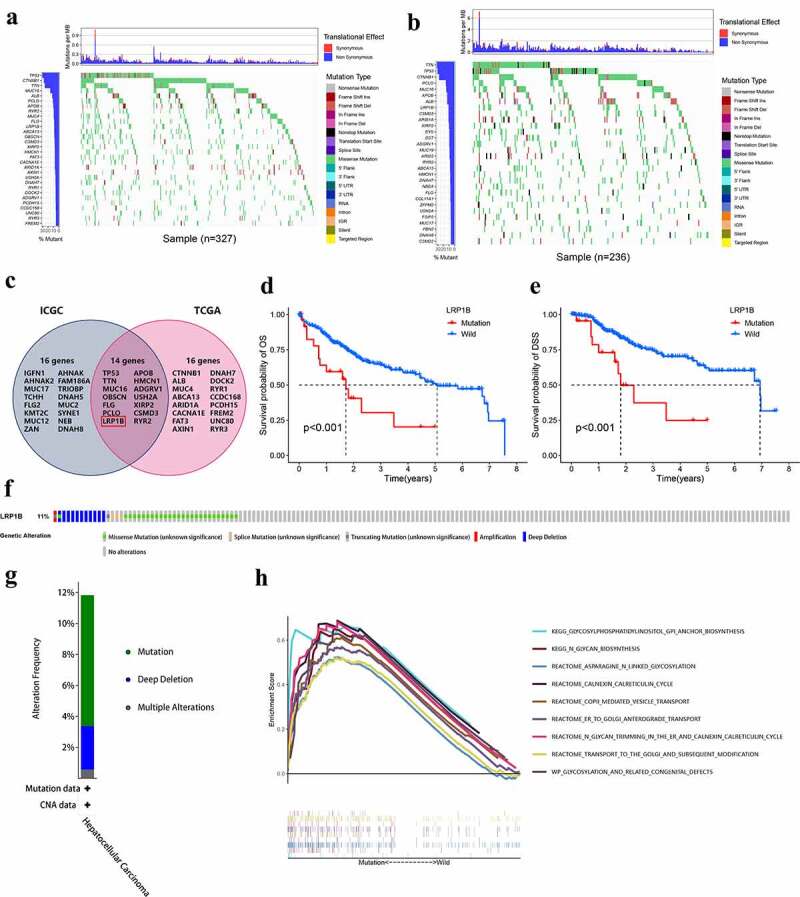
**Genomic alterations of LRP1B are associated with HCC**. The waterfall plot displays the frequently mutated genes in HCC from the TCGA (a) and ICGC cohort (b). The left panel shows the genes ordered by their mutation frequencies. The right panel presents different mutation types. (c) Venn diagram of frequently mutated genes covered by both the TCGA and ICGC cohorts. The K-M survival curves for OS (d) and DSS (e) of HCC with wild-type or mutant LRP1B. (f) OncoPrint summary of alterations on a query of LRP1B. Five types of genetic alterations were defined: missense mutation, splice mutation, truncating mutation, amplification, and deep deletion. (g) Summary of the alteration frequency derived from mutation, deep deletion, and multiple alterations in HCC. (h) GSEA analysis of HCC samples with LRP1B wild-type and mutation from the TCGA cohort.

The top 30 frequently mutated genes in HCC samples from the TCGA and ICGC databases are shown in Figure 7a, b, respectively. The intersection was taken to obtain a gene set with high mutation rates for Chinese and Americans (Figure 7c). *LRP1B* mutation was the sole mutation found to have a significant association with OS and DSS in a vicious direction, indicating that patients with mutated *LRP1B* had a worse prognosis (Figure 7d, e). There was no significant difference in OS between other mutant and wild-type genes (Figure S5). The cBioPortal was employed to identify the frequency of *LRP1B* variations in HCC based on DNA-seq data. Among the 366 HCC patients, 42 (11%) had changes in the *LRP1B*, including missense mutation (7.4%), splicing mutation (0.82%), truncation mutation (0.55%), amplification (0.27%) and deep deletion (3.0%) (Figure 7f, g). GSEA analysis demonstrated that pathways related to protein modification (specifically N-glycosylation) and transport were significantly enriched in samples with *LRP1B* mutation, for instance, N-glycan trimming in the ER and Calnexin/Calreticulin cycle, N-Glycan biosynthesis, and ER to Golgi Anterograde Transport (Figure 7h).

## Discussion

In this study, we probed the expression of *LRP1B* in HCC tissues and cell lines as well as its role in HCC development. Firstly, *LRP1B* was strongly expressed in HCC tissues as compared with adjacent normal tissues based on the analyses of the RNA-seq data in public databases. Furthermore, such differences in expression have been verified in cell lines as well. Bioinformatics analyses indicated that HCC patients with low expression of *LRP1B* had much longer OS than those with high *LRP1B* expression. Subsequently, HCC cells proliferation, migration and invasion abilities were attenuated after siRNA-mediated reduction of *LRP1B*. The efficiency of doxorubicin was enhanced in this same setting. The silencing of *LRP1B* regulated the PERK-ATF4-CHOP signaling pathway to inhibit HCC progression. Additionally, the high mutation frequency of *LRP1B* in HCC tissues was associated with adverse outcomes.

A growing number of studies were conducted to demonstrate the involvement of *LRP1B* in cancer progression and tumor malignancy. *LRP1B*, also known as LRP-DIT (LRP deletion in tumors), was first determined to be frequently inactivated in non-small cell lung cancer (NCLC) cell lines and was considered a putative tumor suppressor [24]. Multiple studies have also evaluated the prognostic value of *LRP1B* mutation in identifying a high-risk cohort. *LRP1B* knockdown is related to worse outcomes for glioblastoma patients [25]. Patients with *LRP1B* mutation were identified to be associated with prolonged survival in both melanoma and NCLC cohorts [8]. Moreover, *LRP1B* is thought to be a marker of TMB to guide immunotherapy [13,14,26]. However, these studies have only conducted multi-omics studies without exploring the specific mechanism.

*LRP1B* activity is commonly downregulated during tumor progression in several tumor types, such as renal and colon cancer. *LRP1B* deletion improves growth, cell migration, and invasion in HEK293 cells [9]. Colon cancer cells’ development and metastasis ability are significantly enhanced after *LRP1B* knockdown through β-catenin/TCF signaling [27]. However, there are few studies on *LRP1B* expression levels and the functions in HCC.

The high expression of *LRP1B* in HCC and the close correlation between overexpression and poor prognosis indicate that *LRP1B* is evidently related to the development of HCC. Undoubtedly, targeting *LRP1B* mRNA with siRNA induced a potent inhibition of the malignant phenotype, including proliferation, colony formation, migration and invasion in HepG2 and HuH-7 cells. These results are consistent with the findings of relevant clinical studies that demonstrated significantly higher *LRP1B* expression in patients of stage II and T2 than those of stage I and T1. This is contrary to the results of previous studies on colon cancer and kidney cancer, wherein the heterogeneity between tumors was a possible factor. The specific reasons require further investigation.

Malignant tumors can proliferate indefinitely [28]. Therefore, we applied a variety of functional experiments to study the contribution of *LRP1B* to the proliferation of HCC cells. The cell viability is significantly reduced after *LRP1B* knockdown using CCK-8 assays. As an analog of thymine, EdU can be integrated into the newly synthesized DNA of replicating cells, which is utilized as a proliferation marker to mark cells in the DNA replication phase [29]. The ratio of such cells is lower in *LRP1B* knockdown cells. Single cells transfected with *LRP1B*-siRNA showed the same results in colony formation, that being, inhibited proliferation. In addition, the migration and invasion ability of HCC cells is significantly suppressed after *LRP1B* deletion. EMT is a critical step in tumor migration and invasion [30]. Therefore, analyzing the expression of essential proteins of EMT clarifies the changes in invasion and migration at the molecular level. The Western blot results confirmed the increase in protein levels of E-cadherin, but N-cadherin and vimentin are decreased in *LRP1B*-downregulated cells instead. Tumor cell migration and invasion are directly associated with metastasis [31]. However, no significant difference in the metastasis status (M stage) of the two groups of patients with high and low expression of *LRP1B* was seen in the clinical correlation analysis. One possible reason is the small number of clinical samples and the inaccurate statistics of M staging. The contribution of *LRP1B* to HCC metastasis requires further in vivo studies.

Chemoresistance is a primary driver of cancer recurrence, leading to a devastating prognosis for HCC patients [32,33]. Doxorubicin is one of the most widely used drugs for chemotherapy in HCC, but the effects of either doxorubicin monotherapy or combination treatment with other chemotherapy drugs remain unfavorable [34,35]. There was a significant decrease in cell viability when treated with doxorubicin after down-regulating *LRP1B*. The cytotoxic effect of doxorubicin on HCC cells is mainly achieved through P53-mediated apoptosis [36]. Bcl-2 and Bax both belong to the Bcl-2 family, where Bcl-2 belongs to the anti-apoptotic gene whereas Bax belongs to the pro-apoptotic gene [37]. Western blot analyses revealed that the pro-apoptotic signals (the ratio of Bax/Bcl-2 proteins) and p53 proteins were both elevated. The above results indicated that the downregulated *LRP1B* can not only reduce the malignancy of HCC but also enhance the sensitivity of the cells to doxorubicin, which may contribute to improved outcomes.

In recent years, the study of the correlation between endoplasmic reticulum stress and cancers has received increasing focus [38–40]. The unfolded protein response caused by endoplasmic reticulum stress regulates cells to adapt to the unfavorable microenvironment whilst playing a vital role in tumor cell growth, differentiation, maintenance of protein homeostasis, and immune response [41,42]. Our study showed that the downregulation of *LRP1B* inhibits tumor development by promoting the activation of the PERK-ATF4-CHOP signaling pathway. The expression of BiP, a molecular chaperone related to endoplasmic reticulum stress, has also been similarly altered. Tumor cells, in response to endoplasmic reticulum stress, upregulate molecular chaperones to restore protein homeostasis in the endoplasmic reticulum, either by promoting protein folding and degradation capabilities or by reducing the need for such effects [43]. This response initially maintains the normal homeostasis of the endoplasmic reticulum through negative feedback but may eventually inhibit cells progression in response to solid or sustained endoplasmic reticulum stress [44]. Hepatocytes and HCC cells are both rich in endoplasmic reticulum, therefore the continuous aggravation of endoplasmic reticulum stress may inhibit the occurrence and development of HCC by inducing HCC apoptosis and other ways. This study showed that the knockdown of *LRP1B* cannot induce apoptosis by activating the endoplasmic reticulum stress. In addition to generating apoptosis pathways, endoplasmic reticulum stress can inhibit tumor cells by enhancing the autophagy of tumor cells or promoting endoplasmic reticulum stress protection. Further studies will be conducted to explore the specific mechanism.

*LRP1B* mutation is closely related to the adverse outcomes and shorter OS of HCC patients. Furthermore, there is no significant difference in immune cell infiltration between these *LRP1B* mutant and wild-type HCC patients. These patients are all treated with chemotherapy rather than immunotherapy, hence immune cell infiltration was not induced [45,46]. Thence, we performed a GSEA enrichment analysis from where we obtained interesting result results. The *LRP1B* mutant samples were enriched in protein glycosylation signaling pathways and closely related to the vesicle transport as well as the modification of the endoplasmic reticulum and Golgi apparatus. Glycosylation modification refers to the process of transferring glycosyl groups to proteins under the action of glycosyl-transferase followed by the formation of glycosidic bonds with amino acid residues on the proteins, of which N-glycosylation is the most crucial type of modification [47]. At present, glycosylation has been widely recognized as an essential hallmark of cancer and significantly correlates with the chemoresistance of tumors [48]. The N-glycosylation inhibitor tunicamycin reduces the efflux of cisplatin in various liver cancer cell lines after downregulating the level of N-glycosylation modification, thereby increasing the sensitivity of liver cancer cells to chemotherapy drugs [49]. Tunicamycin dramatically increases chemotherapy-induced apoptosis by evoking endoplasmic reticulum stress in gastric cancer and ovarian cancer cells through the inhibition of glycosylation [50,51]. Therefore, we suggested that the poor survival of HCC patients with *LRP1B* mutants may be due to glycosylation leading to increased chemotherapy resistance. Simultaneously, *LRP1B* acts as a potential tumor suppressor gene because of its ability to inhibit glycosylation, however, the specific mechanism is not known. We will verify our conjecture and improve the research of related mechanisms in our future phase.

## Conclusions

To summarize, our study revealed that *LRP1B* knockdown inhibits the proliferation, migration, and invasion of HCC cells while enhancing the sensitivity of HCC cells toward doxorubicin. *LRP1B* deletion inhibits HCC progression by activating the PERK-ATF4-CHOP signaling pathway. The high expression and mutation of *LRP1B* are strongly associated with the poor prognosis of HCC patients. These results indicate that *LRP1B* may be a potential prognostic biomarker and effective therapeutic target for HCC patients.

## Supplementary Material

Supplemental Material

## Data Availability

The data that support the findings of this study are openly available in TCGA database (http://www.tcga.org/), GEO database (https://www.ncbi.nlm.nih.gov/geo/), and ICGC database (https://dcc.icgc.org/).
