# Supplementary material for: Downregulation of Low-density lipoprotein receptor-related protein 1B (LRP1B) inhibits the progression of hepatocellular carcinoma cells by activating the endoplasmic reticulum stress signaling pathway
Source: Bioengineered. 2022 Apr 7;13(4):9467–81. doi: 10.1080/21655979.2022.2060778 (PMC9161869; doi:10.1080/21655979.2022.2060778)
Supplement: Supplemental Material [file KBIE_A_2060778_SM2528.docx]

**Supplementary material**

**Supplementary figures**

**
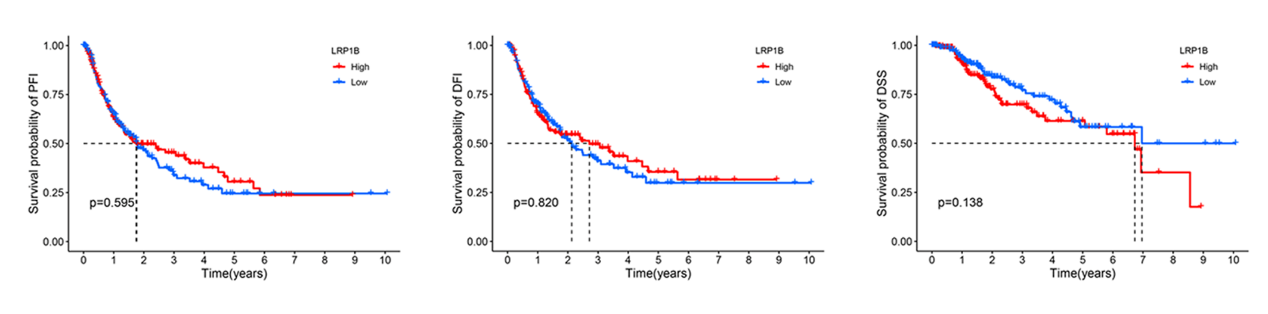
**

**Figure S1. The K-M survival curves for PFI, DFI, and DSS of HCC with low or high LRP1B.**

**
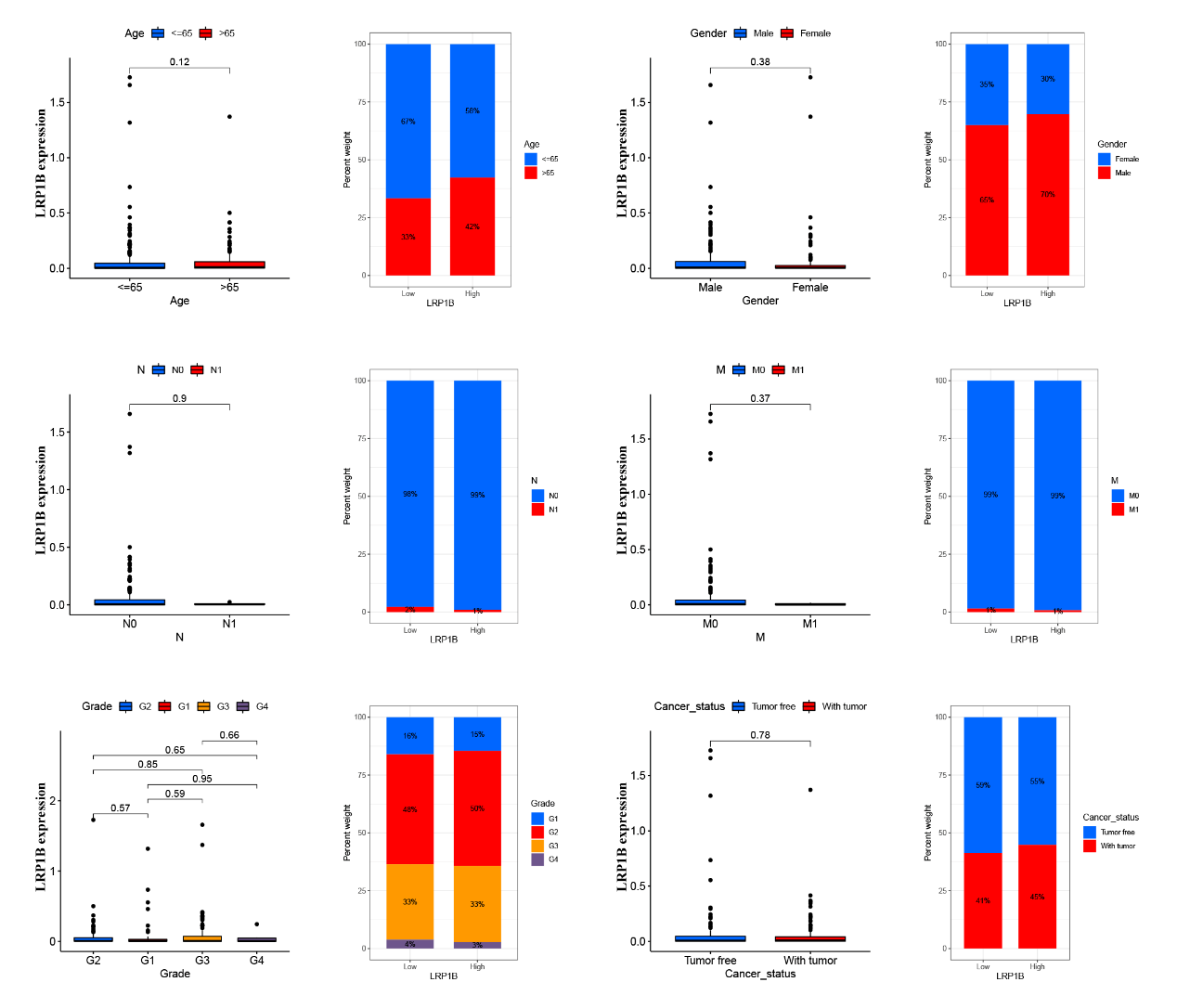
**

**Figure S2. Relationship between LRP1B expression and clinical characteristics in HCC.**

**
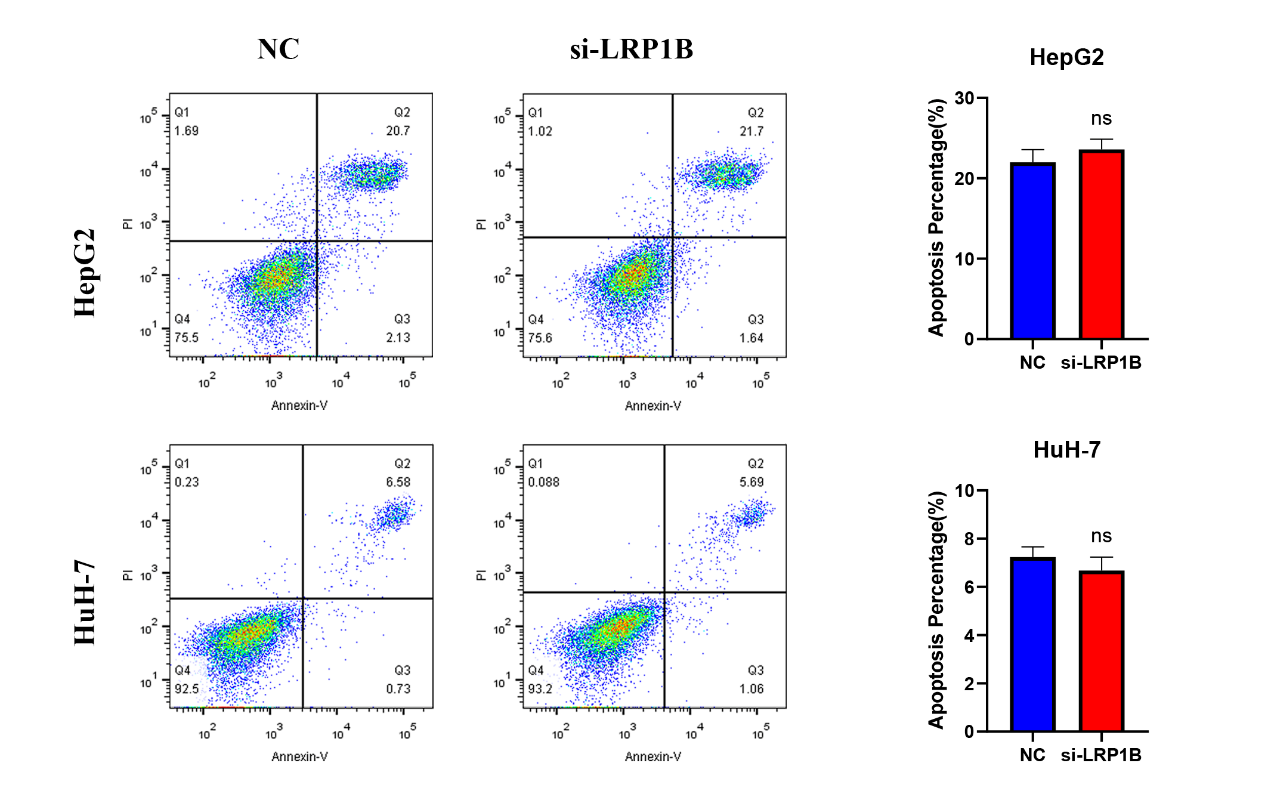
**

**Figure S3. Apoptosis of HCC cells with LRP1B knockdown analyzed by flow cytometry.**

**
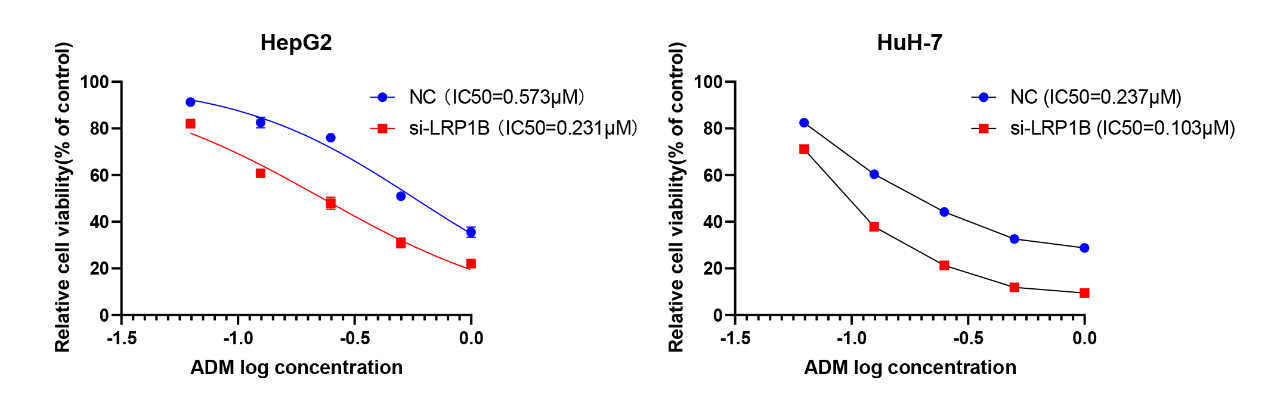
**

**Figure S4. Changes in the sensitivity of HCC to doxorubicin after LRP1B knockout.**

**
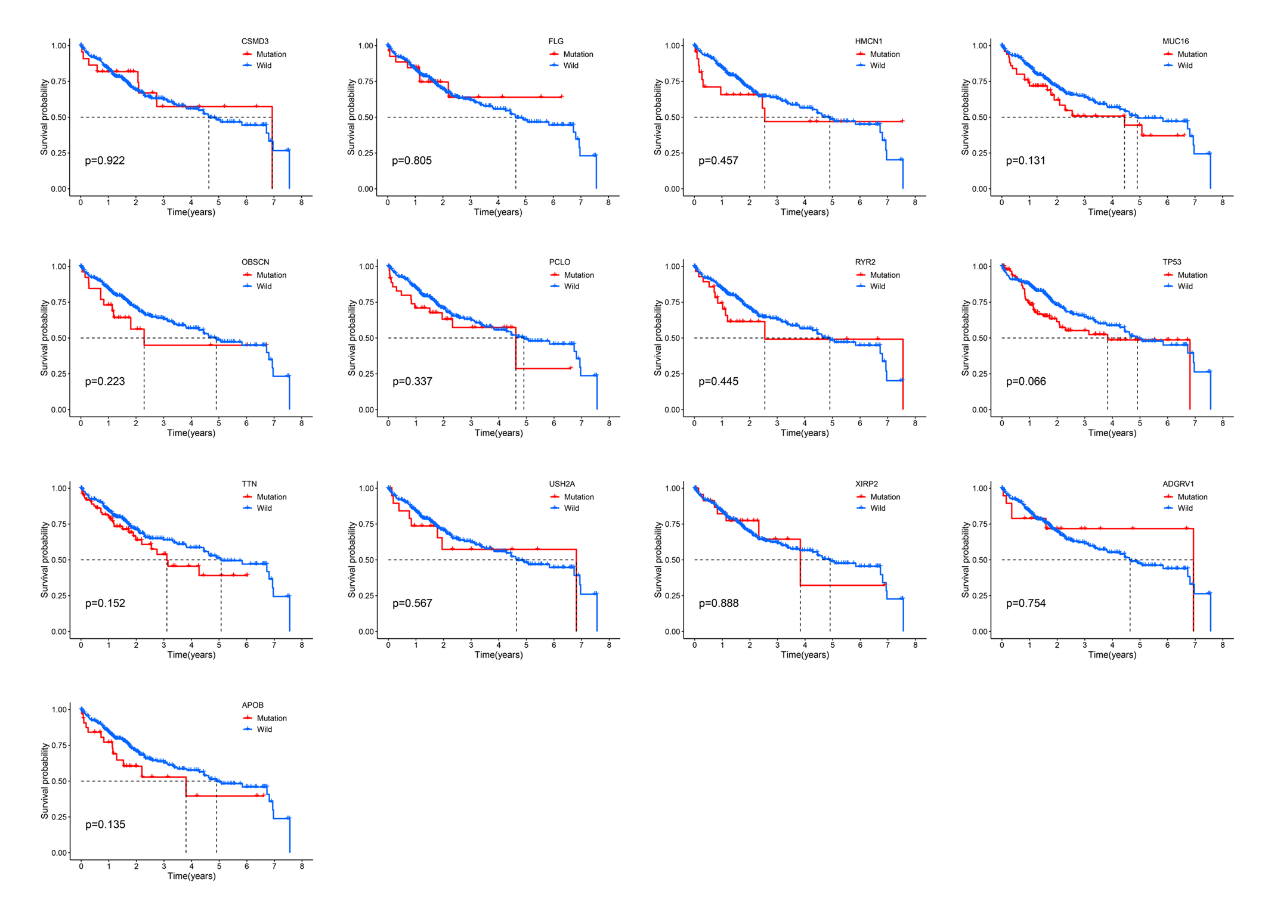
**

**Figure S5. K-M survival analyses of mutations in other genes.**

**Supplementary tables**

**Table S1.** The sequences of siRNA.

| **Name** | **Sequence (5’ → 3’)** |
| --- | --- |
| **LRP1B-siRNA** |  |
| Sense | CCAAGCAUCCUUGUGCUAATT |
| Antisense | UUAGCACAAGGAUGCUUGGTT |
| **NC-siRNA** |  |
| Sense | UUCUUCGAACGUGUCACGUTT |
| Antisense | ACGUGACACGUUCGGAGAATT |

**Table S2.** Details of the datasets included in this analysis

| **Dataset** | **Contributor (s), Year** | **Tumor (n)** | **Normal (n)** | **Platform** |
| --- | --- | --- | --- | --- |
| **TCGA-LIHC** | TCGA | 374 | 50 | Illumina HiSeq |
| **GSE45114** | Wei L et, 2013 | 24 | 25 | GPL5918 |
| **GSE164760** | Llovet JM, 2021 | 53 | 74 | GPL13667 |
